# Preoperative dexamethasone reduces postoperative pain, nausea and vomiting following mastectomy for breast cancer

**DOI:** 10.1186/1471-2407-10-692

**Published:** 2010-12-23

**Authors:** Jorge Gómez-Hernández, Alba Lorena Orozco-Alatorre, Marisela Domínguez-Contreras, Antonio Oceguera-Villanueva, Salvador Gómez-Romo, Andrea Socorro Alvarez Villaseñor, Clotilde Fuentes-Orozco, Alejandro González-Ojeda

**Affiliations:** 1 Breast Tumor Clinic. Oncologic Institute of Jalisco, Health Secretary. Calle Coronel Calderon 715, Colonia El Retiro. Postal code 44280, Guadalajara, Jalisco. México; 2 Research Unit in Clinical Epidemiology, Specialties Hospital, Western Medical Center. Mexican Institute of Social Security. Avenida Belisario Domínguez 1000, Colonia Independencia. Postal code 44340, Guadalajara, Jalisco. México

## Abstract

**Background:**

Dexamethasone has been reported to reduce postoperative symptoms after different surgical procedures. We evaluated the efficacy of preoperative dexamethasone in ameliorating postoperative nausea and vomiting (PONV), and pain after mastectomy.

**Methods:**

In this prospective, double-blind, placebo-controlled study, 70 patients scheduled for mastectomy with axillary lymph node dissection were analyzed after randomization to treatment with 8 mg intravenous dexamethasone (*n *= 35) or placebo (*n *= 35). All patients underwent standardized procedures for general anesthesia and surgery. Episodes of PONV and pain score were recorded on a visual analogue scale. Analgesic and antiemetic requirements were also recorded.

**Results:**

Demographic and medical variables were similar between groups. The incidence of PONV was lower in the dexamethasone group at the early postoperative evaluation (28.6% *vs*. 60%; *p *= 0.02) and at 6 h (17.2% *vs*. 45.8%; *p *= 0.03). More patients in the placebo group required additional antiemetic medication (21 *vs*. 8; *p *= 0.01). Dexamethasone treatment significantly reduced postoperative pain just after surgery (VAS score, 4.54 ± 1.55 *vs*. 5.83 ± 2.00; *p *= 0.004), at 6 h (3.03 ± 1.20 *vs*. 4.17 ± 1.24; *p *< 0.0005) and at 12 h (2.09 ± 0.85 *vs*. 2.54 ± 0.98; *p *= 0.04). Analgesics were required in more patients of the control group (21 *vs*. 10; *p *= 0.008). There were no adverse events, morbidity or mortality.

**Conclusions:**

Preoperative intravenous dexamethasone (8 mg) can significantly reduce the incidence of PONV and pain in patients undergoing mastectomy with axillary dissection for breast cancer.

**Trial registration number:**

NCT01116713

## Background

Breast cancer is the most frequent malignant neoplasm worldwide. In emerging countries such as Mexico, there has been an increase in its frequency and mortality [1,2] and it is the second most frequent neoplasm after cervical carcinoma. Between 25,000 and 30,000 new cases are diagnosed annually. Unfortunately, only a few women have regular mammography screening so the proportion of patients with locally advanced disease at diagnosis is high. In 2003, only 5-10% of newly diagnosed cases in Mexico were clinical stages 0 or I [2]. Surgical resection with axillary lymph node dissection constitutes the treatment of choice associated with neoadjuvant therapy and postoperative chemotherapy and/or radiation therapy.

Postoperative nausea and vomiting (PONV) are the most common complications after anesthesia and surgery [3,4]. Women undergoing mastectomy with axillary dissection are at a particularly high risk for the development of PONV and an incidence of 60-80% in patients receiving no antiemetic has been reported [5-7]. Emetic episodes predispose to aspiration of gastric contents, wound dehiscence, psychological distress, and delayed recovery and discharge times [3]. These justify the use of prophylactic antiemetics in women scheduled for mastectomy. Most of the currently used antiemetics, including antihistamines, butyrophenones and dopamine receptor antagonists have been reported to cause occasional undesirable adverse effects, such as excessive sedation, hypotension, dry mouth, dysphoria, hallucinations and extrapyramidal signs [3]. Antiserotonins (e.g., ondansetron) are available for the prevention and treatment of PONV in patients undergoing various types of surgery [4]. However, the use of prophylactic antiemetic therapy with antiserotonins has been criticized for being too expensive [8,9].

Dexamethasone was first reported to be an effective antiemetic regimen in patients receiving cancer chemotherapy [10]. Holte et al. [11] have reviewed the available randomized trials (1996-2001) regarding perioperative single-dose steroid administration and found that dexamethasone had antiemetic and analgesic effects in various types of operations. Wattwil et al. [12] showed that dexamethasone at 4 mg was effective for the prevention of PONV following breast surgery but they could not demonstrate any difference in postoperative pain.

The purpose of this study was to evaluate the efficacy of dexamethasone treatment for reducing pain and PONV as well as analgesic and antiemetic requirements in women undergoing general anesthesia for mastectomy with axillary lymph node dissection.

## Methods

### Patients

Between June and August 2009, 70 patients undergoing mastectomy with axillary dissection were studied in a prospective, randomized, double-blind clinical trial. Patients were randomized to receive intravenous dexamethasone (8 mg) or homologated placebo 60 minutes before skin incision, using an equal number of blinded envelopes. Patients of American Society of Anesthesiologists classes III and IV were excluded. Further exclusion criteria were age more than 80 years; pregnancy; active menstruation; treatment with steroids; severe diabetes mellitus (serum HbA1c > 8%); use of opioids, sedatives or any kind of analgesics less than one week before mastectomy, or a history of alcohol or drug abuse. Patients with any history of motion sickness and/or previous PONV after any surgical procedure were excluded. All patients were admitted to the hospital one day before the operation and were followed from hospital admission until 30 days after the surgical procedure to detect any medical or surgical morbidity.

### Anesthesia and surgery

All patients underwent a standardized general anesthesia procedure and none of them received any preanesthetic medication. Induction used intravenous propofol (2 mg/kg body weight) and fentanyl (3-5 μg/kg). Vecuronium bromide (0.1 mg/kg) was used to facilitate tracheal intubation. Anesthesia was maintained with 2-3% sevoflurane and 66% nitrous oxide in oxygen. Ventilation was controlled mechanically and maintained constant throughout surgery using an anesthetic and respiratory gas analyzer for monitoring.

All patients were monitored with indirect determinations of arterial pressure and heart rate using standard techniques, as well as the expired CO_2 _content and oxygen blood saturation. Afterwards, all patients were extubated and transferred to the immediate postsurgical care unit with cardiovascular and oxygen monitoring.

### Surgical procedures

All patients were treated according to the preoperative clinical stage with radical mastectomy or breast conservative surgery with lymph node axillary dissection by the same surgical team. In all patients, closed suction drains were placed in the region subjected to surgery and were removed during the following days. Chemotherapy and/or radiotherapy were administered 3-4 weeks after uncomplicated surgical resections.

### Analgesia and antiemetic therapies

Pain was assessed immediately on return to the recovery room and at 6, 12 and 24 h after the operation using a visual analogue scale (VAS; 0 = no pain to 10 = most severe pain). Analgesia was given as intravenous sodium ketorolac (30 mg every 8 hours) and intravenous tramadol infusion (50 mg) was used as a backup analgesic medication. The incidence of PONV was recorded immediately on return to the recovery room and at 6, 12 and 24 h after the operation, using a three point ordinal scale (0 = none, 1 = nausea, 2 = retching, 3 = vomiting). Nausea was defined as a subjectively unpleasant sensation associated with awareness of the urge to vomit, retching was defined as the labored, spasmodic, rhythmic contraction of the respiratory muscles without the expulsion of gastric contents, and vomiting was defined as the forceful expulsion of gastric contents from the mouth. Intravenous ondasetron (4-8 mg) was given for antiemetic treatment on demand.

### Data collection and statistical analysis

Postoperative complications were recorded during hospitalization and the patients were followed up to 30 days after discharge. Additional data collected included patient age, body mass index (BMI), any history of smoking and neoadjuvant chemotherapy, anesthesia and operation time and the frequency of use of analgesic and antiemetic drugs. These parameters were summed and compared between the dexamethasone and placebo groups. The study endpoints were postoperative nausea and vomiting, and pain measured by the VAS and the need for additional analgesic and antiemetic drugs.

The sample size was predetermined. We expected a 35 percent point difference in the incidence of nausea and vomiting between groups favoring dexamethasone use. The α error was set at 0.05 and β error at 0.20; *n *= 35 patients for each group was considered adequate, according to a power analysis. Results are expressed as percentages and as the mean ± standard deviation (SD). Student's *t *test, and Fisher's Exact Test were used for the analysis of quantitative and qualitative data, respectively. Two-sided tests were used to declare statistical significance at *p *< 0.05.

### Ethical considerations

The study was conducted according to the principles of the Declaration of Helsinki of 1989 and the Mexican Health Guidelines. The Ethical and Research Committees of the Oncologic Institute of Jalisco, Mexico approved all protocols. Full, written informed consent was obtained from all patients before their inclusion in the study.

## Results

**Figure 1:**
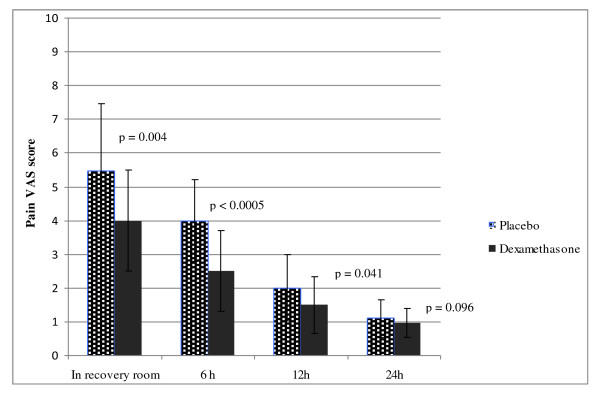
**Pain VAS score of patients in the control and study groups**.

There were no significant differences between the two groups with regard to mean age, weight, height, BMI, neoadjuvant chemotherapy, type of surgical procedure, length of surgical procedures, length of anesthesia or operative bleeding (Table 1). Figure 1 shows the median VAS values for pain. Dexamethasone significantly reduced postoperative pain just after surgery (VAS score, 4.54 ± 1.55 *vs*. 5.83 ± 2.00; *p *= 0.004), 6 h after the operation (VAS score, 3.03 ± 1.20 *vs*. 4.17 ± 1.24; *p *< 0.0005) and 12 h after the breast surgery (VAS score, 2.09 ± 0.85 *vs*. 2.54 ± 0.98; *p *= 0.04). No difference was observed 24 h after the operation (1.23 ± 0.42 *vs*.1.43 ± 0.55; *p *= 0.09). Analgesics were required in more patients of the control group than in the dexamethasone group (21 *vs*. 10; *p *= 0.008). The mean dose of intravenous tramadol was lower in the study group (36.01 ± 12.62 *vs*. 55.74 ± 27.36; *p *= 0.03).

**Table 1:** Baseline characteristics of the patients in the study and the placebo groups.

	Placebo group (n = 35)	Dexamethasone group (n = 35)
Age (years)^a^	49.89 ± 10.58	50.11 ± 12.37
Weight (kg)^a^	67.94 ± 9.91	69.52 ± 13.63
Height (cm)^a^	158.14 ± 6.33	157.31 ± 6.57
Body mass index^a^	27.20 ± 4.29	28.20 ± 6.38
Normal weight 20-25 (***%***)	11 (31.4%)	12 (34.3%)
Overweight 25.1-29.9 (***%***)	15 (42.9%)	14 (40%)
Obesity grade I 30-34.9 (***%***)	7 (20%)	5 (14.3%)
Obesity grade II 35-39.9 (***%***)	2 (5.7%)	2 (5.7%)
Obesity grade III >40 (%)	0	2 (5.7%)
Smoking (***%***)	2 (5.7%)	4 (11.4%)
Neoadjuvant chemotherapy (***%***)	13 (37.1%)	15 (42.8%)
Surgical procedure		
Radical mastectomy + axillary dissection (***%***)	32 (91.4%)	34 (97.1%)
Quadrantectomy + axillary dissection (***%***)	3 (8.6%)	1 (2.9%)
Surgery time (min)^a^	119.42 ± 26.97	120.54 ± 30.34
Length of anesthesia (min)^a^	140.71 ± 26.76	139.60 ± 34.56
Bleeding (ml)^a^	165.85 ± 61.10	156.85 ± 60.11

^a^Values are expressed as the mean ± standard deviation.

The incidence of PONV is described in Table 2. There were significant differences between the two groups in terms of the early postoperative evaluation in the recovery room and at 6 h, favoring the dexamethasone group. More patients in the placebo group required the administration of additional antiemetics (21 *vs*. 8; *p *= 0.01); the mean dose of ondansetron was 7.05 ± 2.80 mg in the control group and 4.50 ± 1.41 mg in the study group (*p *= 0.02).

**Table 2:** Incidence of PONV in the dexamethasone and placebo groups

	Placebo group (n = 35)	Dexamethasone group (n = 35)	P
In recovery room:			
➢ PONV	21 (60%)	10 (28.6%)	0.02
➢ Asymptomatic	14 (40%)	25 (71.4%)	
6 h after surgery:			
➢ PONV	16 (45.8%)	6 (17.2%)	0.03
➢ Asymptomatic	19 (54.2%)	29 (82.8%)	
12 h after surgery:			
➢ PONV	6 (11.2%)	4 (11.5%)	0.35
➢ Asymptomatic	29 (82.8%)	31 (88.5%)	
24 h after surgery:			
➢ PONV	5 (14.3%)	1 (2.9%)	0.23
➢ Asymptomatic	30 (85.7%)	34 (97.1%)	

PONV, postoperative nausea and vomiting

*p *values calculated using two-sided Fisher's Exact Test.

The hospital stay was 1-3 days. No morbidity was observed during the follow up period. Drains were removed when the fluid output was less than 50 ml/24 h. One patient in the control group developed a generalized rash before the skin incision was started. The adverse event was treated with intravenous antihistaminic and hydrocortisone with complete resolution. This case was excluded and substituted with a new candidate. There was no mortality.

## Discussion

In this randomized, double-blind, placebo-controlled study, preoperative dexamethasone (8 mg) significantly reduced the incidence of pain, PONV and analgesic and antiemetic requirements after breast surgery for cancer. PONV is an unpleasant, distressing and exhausting complication for patients. It can prolong recovery time, delay patient discharge and increase hospital costs [13]. The etiology of PONV after breast surgery is not entirely clear. Factors affecting PONV after breast surgery are those related to the patients, those related to the surgical procedure and the anesthetic technique and the postoperative care [14]. Risk-scoring systems have been proposed based on logistic regression modeling to select patients for prophylaxis against PONV [15,16]. Complicated formulations were simplified establishing four predictor factors: a) female sex; b) a history of motion sickness and/or PONV; c) absence of a smoking habit and d) any use of opioids [17]. If none, one, two, three or four of these factors were present, the incidence of PONV was 10%, 23%, 39%, 61% and 79% respectively [17]. Breast surgery inherently caters to high-risk patients and being female was an independent predictor for PONV in multivariate analysis [15,16]. In our study patients with history of motion sickness and/or PONV were not included and only a few of our patients were active smokers. The first condition lowers the threshold for vomiting [18] and smoking provides some protection against it [3]. The length of the surgical procedure also increases the risk of PONV. As established by Sinclair et al. [16], each 30 min increase in the duration of surgery increases the incidence of PONV by 60%. Anesthetic-related factors have a strong influence on the incidence of PONV because the use of opioids as premedications or to maintain anesthesia stimulates central nervous system (CNS) opioid receptors [19]. Nitrous oxide causes PONV by stimulating the CNS with catecholamine release and changes in middle-air pressure with subsequent stimulation of the vestibular system and elevation of abdominal pressure as a consequence of the exchange of nitrous oxide and nitrogen in gas introduced into the gastrointestinal tract during mask ventilation [20-24]. The use of propofol for maintaining anesthesia has a positive effect on reducing PONV [25]. Postoperatively, the administration of opioids might contribute to these episodes [26].

Since the original description of dexamethasone as an effective antiemetic drug [10], studies have reported it to be effective for the prevention of nausea and vomiting after different surgical procedures. The biological action of a glucocorticoid begins 1-2 h after administration [27]. The antiemetic mechanism of dexamethasone is not known. However, central inhibition of prostaglandin synthesis, inhibition of endogenous opioid release and changes in the permeability of the blood-brain barrier to serum proteins have been suggested [27,28]. The analgesic effects of glucocorticoids are mainly provided through the peripheral inhibition of phospholipase, thereby decreasing the products of the cyclooxygenase and lipoxygenase pathways in the inflammatory response [29].

In this trial, a variety of risk factors were present in both groups of patients. This included being female, nonsmoking, receiving neoadjuvant chemotherapy, undergoing an operative procedure and anesthesia, the presence of postoperative pain and the use of opioids. All of these factors are considered to affect the incidence of PONV; however, they were well balanced between the placebo and dexamethasone treatment groups. Therefore, the difference in the rate of patients experiencing PONV among the groups can be attributed to the use of dexamethasone.

Our results are similar to those reported by Fujii and Nakayama [30]. They designed a randomized clinical trial with 90 patients submitted to breast surgery (24% of them treated without axillary lymph node dissection). They divided the patients into three groups of 30. Patients received intravenous dexamethasone at doses of 4 or 8 mg preoperatively and the control group received placebo. They evaluated the incidence of PONV and analgesic requirements until 24 h after the surgical procedure. The incidence of PONV was 67% in the placebo group versus 33% and 27% in the 4 mg and 8 mg dexamethasone groups (*p *= 0.01 and 0.002, respectively). The need for rectally administered indomethacin for relieving intolerable pain was less in patients who had received 8 mg dexamethasone than in those who received placebo (*p *= 0.001) or dexamethasone at 4 mg (*p *= 0.034). No difference in analgesic requirement was found between the 4 mg dexamethasone and placebo groups (*p *= 0.18). They concluded that dexamethasone at 8 mg effectively decreased PONV and analgesic requirements in women undergoing general anesthesia.

## Conclusions

Our results suggest that preoperative dexamethasone at 8 mg ameliorates nausea, vomiting, pain and reduces the analgesic and antiemetic requirements of women after breast surgery for cancer without apparent side effects. It appears to be a valuable treatment for preventing such adverse postoperative symptoms.

## Competing interests

The authors declare that they have no competing interests.

## Authors' information

Breast Tumor Clinic. Oncologic Institute of Jalisco, Health Secretary. Calle Coronel Calderon 715, Colonia El Retiro. Postal code 44280, Guadalajara, Jalisco. Mexico.

Research Unit in Clinical Epidemiology, Specialties Hospital, Western Medical Center. Mexican Institute of Social Security. Avenida Belisario Dominguez 1000, Colonia Independencia. Postal code 44340, Guadalajara, Jalisco. Mexico.

## Authors' contributions

ASAV, CFO and AGO were responsible for conceiving and designing the study as well as analyzing and interpreting the data and writing the different stages of the manuscript. ALOA and MDC were responsible for coordination of the subject recruitment for the study and participated in data interpretation. JGH, AOV and SGR were responsible to treat surgically the study patients.

All authors read and approved the final manuscript.

## Pre-publication history

The pre-publication history for this paper can be accessed here:

http://www.biomedcentral.com/1471-2407/10/692/prepub
